# Electrokinetic droplet transport from electroosmosis to electrophoresis

**DOI:** 10.1039/c8sm01788c

**Published:** 2018-11-16

**Authors:** Andrei Bazarenko, Marcello Sega

**Affiliations:** a University of Vienna, Faculty of Physics , Boltzmanngasse 5 , 1090 Vienna , Austria; b Helmholtz Institute Erlangen-Nürnberg , Fürtherstr. 248 , 90429 Nürnberg , Germany . Email: m.sega@fz-juelich.de

## Abstract

Droplet transport in microfluidic channels by electrically induced flows often entails the simultaneous presence of electroosmosis and electrophoresis. Here we make use of coupled lattice-Boltzmann/molecular dynamics simulations to compute the mobility of a droplet in a microchannel under the effect of an external electric field. By varying the droplet solvation free energy of the counterions released at the channel walls, we observe the continuous transition between the electroosmotic and electrophoretic regime. We show that it is possible to describe the mobility of a droplet in a unified, consistent way, by combining the theoretical description of the electroosmotic flow with, in this case, the Hückel limit of electrophoresis, modified in order to take into account the Hadamard–Rybczynski droplet drag.

## Abstract 2

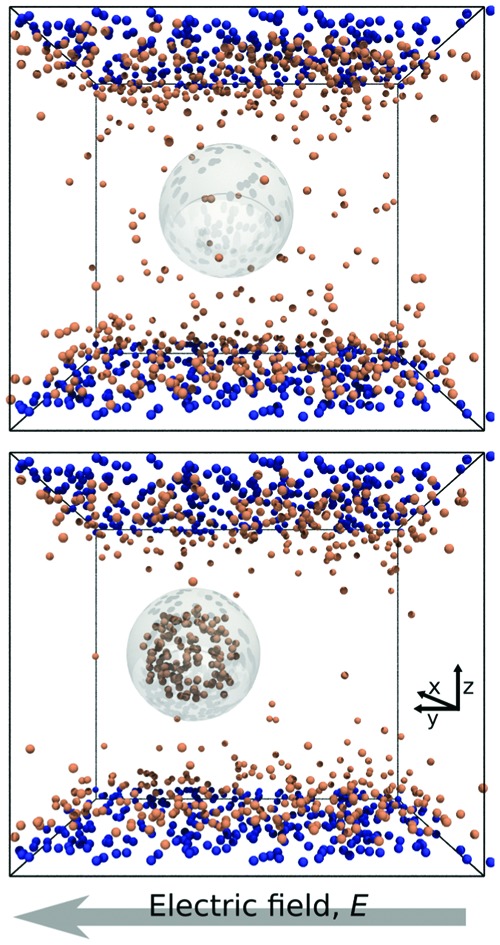
Droplet transport in microfluidic channels by electrically induced flows often entails the simultaneous presence of electroosmosis and electrophoresis.

## 1 Introduction

The ability to manipulate and transport droplets in a controlled fashion is one of the central technological assets in modern microfluidics. Droplet-based microfluidic devices^1^ are using small amounts of liquids, typically in the range from micro- to picoliters, in the form of a binary immiscible liquid mixture at low Reynolds numbers, forming a dispersed component (the droplets) carried in a continuum component (the medium). Dealing with such small amounts of liquids allows fast and controlled mixing thanks to the advantageous dimensional scaling. Individual control over the droplets makes them cheap and viable microreactors^2^ that can be transported and analysed selectively^3^ or in parallel,^4^ allowing to achieve higher throughput than in continuous phase microfluidics.

Droplets can be moved around in microfluidics devices using a variety of techniques,^5^ among which electrokinetic approaches like electroosmosis,^6^,^7^ electrophoresis,^8^–^10^ dielectrophoresis,^11^,^12^ or induced-charge electrokinetic effects^13^ have proven to be very popular. Electrokinetic phenomena, as opposed to electrohydrodynamics ones,^14^ are characterised by the presence of local charge imbalances in the fluid due to the presence of a counter-ions cloud (the so-called diffuse layer) that screens surface charges. A flow can be then generated by acting with an external electric field on the diffuse layer of the continuum phase or of the droplet, in the case of electroosmosis and in the case of electrophoresis respectively.

The distinction between electrokinetics and electrohydrodynamics, similarly to the one between electroosmosis and electrophoresis, has mostly a historical, rather than a physical justification. In practice, electrophoresis and electroosmosis often appear simultaneously. In the case of colloidal electrophoresis, for example, this complicates the correct evaluation of different contributions to the transport properties and, in turn, the measurement of the colloidal charge itself.^15^,^16^ Droplet electrophoresis represents an even more complex problem as in principle counterions can permeate the droplet.

From the computational point of view, following Rotenberg and Pagonabarraga,^17^ one could classify different computational approaches based on whether the solvent and/or the ions are treated explicitly or implicitly. Complex flows in microfluidic channels have been often performed at the mesoscale^18^–^27^ using numerical approaches like the lattice Boltzmann method,^28^ kinetic theory approaches,^29^,^30^ dissipative particle dynamics^31^,^32^ or the multi-particle collision dynamics^33^–^36^ methods, which allow, at the expense of microscopic detail, to overcome the size limitation of atomistic investigations, which are still limited to the nanoscale.^37^–^40^ Regarding the electrophoresis of charged droplets in an electrolyte solution, the theoretical predictions of Ohshima and coworkers^41^ have been compared to numerical approaches, for example, using control volume techniques.^42^

The problem of the electroosmotic contribution to the droplet motion in nanochannels, to the best of our knowledge, has not been addressed so far. In principle, a recently proposed approach for the solution of the Nernst–Plank equations using the lattice-Boltzmann method^27^ could represent a viable and efficient method to investigate this issue using continuum ionic distributions.

In this study, we employ a molecular dynamics/lattice-Boltzmann coupling method to investigate the continuous transition between the purely electroosmotic transport regime and the electrophoretic-dominated one, for a droplet confined in a slit pore. We explore the transition between these two regimes by systematically varying the solvation free energy of counterions in the dispersed phase.

## 2 Methods

We use an implementation of the bicomponent lattice-Boltzmann method of Shan and Chen,^43^ which solves the fluctuating hydrodynamics of the fluid, extending also the Guo forcing term^44^ to the bicomponent case.^45^ The lattice-Boltzmann equations are coupled to the molecular dynamics simulation of point-like ions.^45^,^46^ All simulations are performed using an in-house modified version of the ESPResSo^47^,^48^ simulation package that includes a CPU version of the bicomponent lattice Boltzmann code.

Our model consists of a cubic simulation box of size 48 × 48 × 48 lattice units Δ*x* with two planar walls of surface area *S* = 48 × 48 Δ*x*^2^ each positioned so that the distance between the hydrodynamic no-slip planes is *d* = 42Δ*x*. This grid resolution has been previously shown to be enough to reproduce the analytical results of the ion distribution and of the velocity field for the electroosmotic flow of a single-component fluid.

The fluid has two components, *a* and *b*, with total density *ρ* = *ρ*_*a*_ + *ρ*_*b*_ = 5.0/Δ*x*^3^ and barycentric velocity **u**. We solve the fluctuating hydrodynamic equations of two uncharged fluids with same dielectric permittivity (dielectrophoretic forces are not taken into account)1
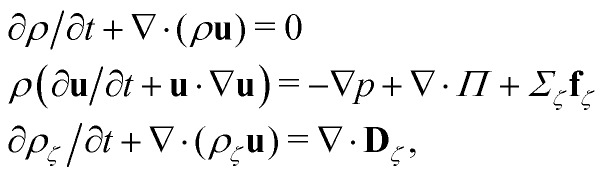
where the forcing term **f**_*ζ*_ that governs the interaction between the fluid components is2
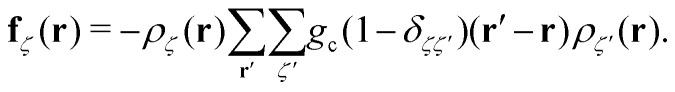



In the equations above, the coupling parameter *g*_c_ = 0.8Δ*x*^3^/Δ*t*^2^, where *ζ* and *ζ*′ indices for the fluid components, *δ*_*ζζ*′_ is the Kronecker symbol, **r** identifies the position of a node and **r**′ loops over adjacent nodes, so that the term (**r**′ – **r**)*ρ*(**r**′) represents a discretised gradient of the density. In addition, *p* is the pressure, and *Π* and **D**_*ζ*_ are, respectively, the stress tensor and diffusion current, whose fluctuating parts obey the corresponding fluctuation-dissipation theorem.^45^ In the present simulation scheme all non-conserved modes are relaxed independently and, in particular, the shear stress and interspecies diffusion relaxations are governed by the kinematic viscosity of the two fluid species, *ν*_*a*_ = *ν*_*b*_ = 40Δ*x*^2^/Δ*t*, and by the interspecies mobility *M* = Δ*t*/6, respectively. The dispersed phase has the volume fraction *φ* ≃ 0.04 of the total fluid and the form of a spherical droplet with radius *R* = 11.2 Δ*x*, calculated as the distance from the centre of the droplet to the shear surface. The thickness *ξ* of the interface is *ξ* ≃ 5Δ*x* and is therefore not within the sharp interface limit.^49^ The no-slip hydrodynamic boundary condition is imposed at the surface of the walls and is implemented as a bounce-back^50^ with tuneable wettability feature.^51^,^52^

Each pair (*i*,*j*) of charges interacts *via* the Coulomb potential *U*_c_ = *k*_B_*T*𝓁_B_*q*_*i*_*q*_*j*_/*r* with *q*_*i*_ being the valency of the *i*-th particle. The characteristic ratio of the energy of thermal fluctuations and the electrostatic energy between two particles is given by the Bjerrum length 𝓁_B_ = *e*^2^/(4π*ε*_0_*ε*_r_*k*_B_*T*), where *ε*_0_ is the dielectric permittivity of vacuum, *ε*_r_ is the relative dielectric constant, *e* the elementary charge and *k*_B_*T* is the thermal energy. We set *l*_B_ = Δ*x* with no dielectric contrast between the fluid components. The two walls are located at *z*_B1_ = 2Δ*x* and *z*_B2_ = 44Δ*x* and are decorated with 576 immobile, pointlike ions each with *q* = –1, thus bearing a surface charge density *σ*_e_ = –0.125*e*/Δ*x*^2^, corresponding to the Gouy–Chapman length 𝓁_GC_ = *e*/(2π*l*_B_*σ*_e_) ≃ 1.27Δ*x*.

The same number of pointlike counterions with opposite valency *q* = 1 are free to access the slit pore volume and therein confined by a Weeks–Chandler–Anderson potential *βU*_WCA_ = 4*ε*[(*σ*/*z*)^12^ – (*σ*/*z*)^6^ + 1/4] for *z* < *σ*2^1/6^, and zero otherwise, which depends on the distance *z* of the ions to each of the walls. The wall-ions interaction (*ε* = 50*k*_B_*T*, *σ* = Δ*x*) prevents positive and negative ions to collapse on each other. The initial distribution of counterions is taken from an equilibrated single-component simulation.

The electrostatic coupling parameter *Ξ* = (*eq*)^2^*l*_b_/*l*_GC_ = 2π*l*_B_^2^*σ*_e_ ≃ 0.8 and the ratio of the wall distance to the average ion distance on the plate is about 15; therefore, the system is in the weak coupling regime.^53^,^54^ Strictly speaking, since no salt is present in the system, the Debye screening length cannot be defined; instead, a screening constant *κ* from the Poisson–Boltzmann solution of the charged walls can be defined as 
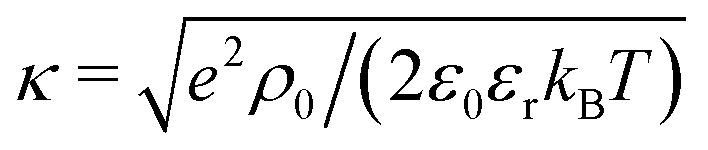
, where *ρ*_0_ is the charge density in the middle of the channel. Using the approximation 
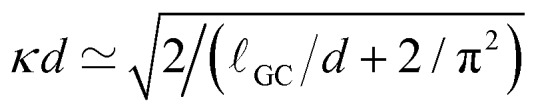
,^55^ one can estimate *κ* ≃ 0.07/Δ*x*. Hence, one can define a reduced screening length with respect to the droplet radius as *δ* = 1/(*κR*) ≃ 1.28. We apply an electric field of strength *E*, ranging from 0.05 to 1.0*k*_B_*T*/(*e*Δ*x*); This quantity should be compared to the potential drop equivalent of the thermal energy (*k*_B_*T* ≃ 25 meV at room temperature) across the droplet, which defines a reduced electric field strength E* = *eER*/*k*_B_*T*.

We apply periodic boundary conditions along the *x* and *y* directions (parallel to the slit pore), taking into account the long-range electrostatic interaction between periodic copies in the *xy* plane, using the electrostatic layer correction (ELC)^56^,^57^ modification of the P3M algorithm.^58^ The lattice-Boltzmann simulation is coupled to the molecular dynamics simulation according to the scheme of Ahlrichs and Dünweg^59^ by integrating with timestep Δ*t* = 0.01 a modified Langevin equation:

3 *m***a**_*i*_ = **F**_*i*,ext_ – *γ*[**v**_*i*_ – **u**(**r**_*i*_)] + **F**_*i*,R_ + **F**_*i*,ps_,

where *γ* = 10/Δ*t* is a bare friction coefficient, **F**_*i*,R_ is a stochastic term with zero mean and variance is a stochastic term with zero mean and variance 〈**F**_*i*,R_(*t*)·**F**_*j*,R_(*t*′)′)〉 = 6 = 6*k*_B_*Tγδ*_*ij*_*δ*(*t* – *t*′). The momentum transferred from the fluid to the particles *via* the viscous coupling in eqn (3) is then redistributed back to the fluid to ensure momentum conservation using a trilinear interpolation.^46^**F**_*i*,ext_ represents the external forces acting on the *i*-th ion, namely, the electric force *eq*_*i*_**E** and the force from the repulsive walls. **F**_ps_ is the particle solvation force, which models the solvation free energy of counterions by acting on particles depending on the gradient of the dispersed phase density

4 **F**_ps_ = –Δ*G*∇*ρ*_a_.

We use Δ*G* to denote the work needed to bring one particle from the middle of the droplet component deep into the other phase. Far from the interface the density is basically constant, and there the ions do not feel any attraction/repulsion by the solvation forces.

## 3 Results

**Fig. 1:**
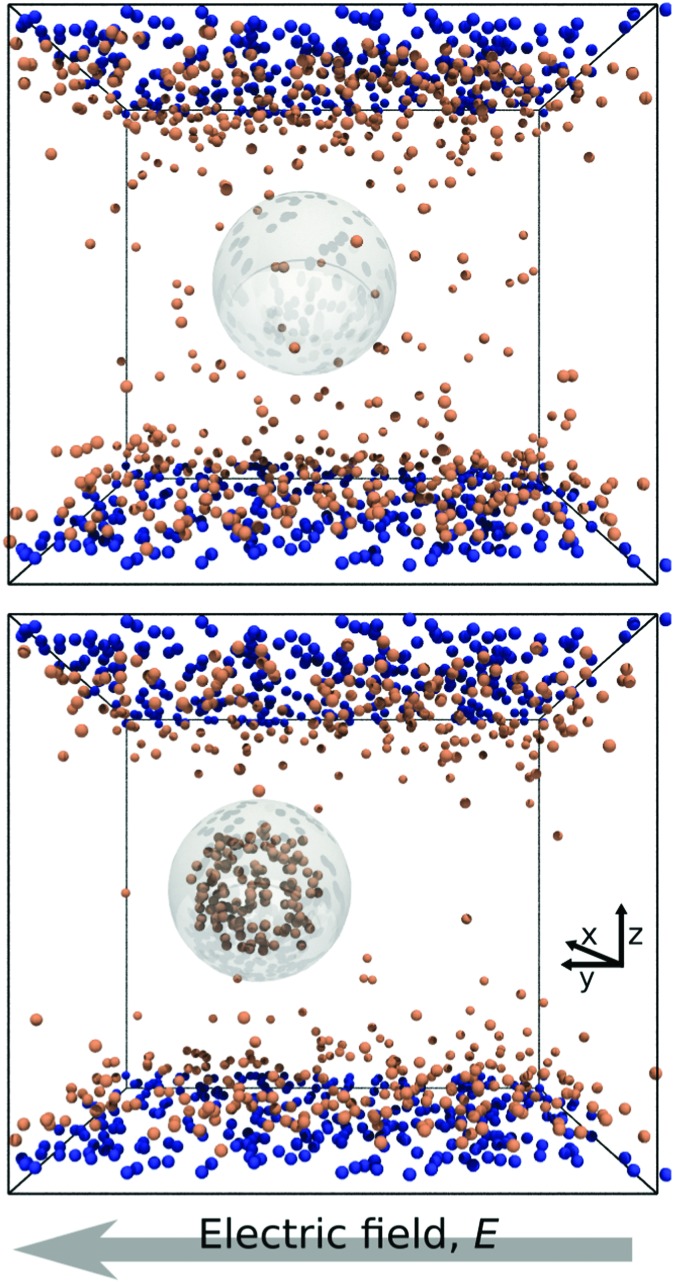
Two snapshots of the system. The grey outline marks the surface of the droplet, while the orange and the blue spheres represent mobile and surface ions, respectively. Top: Δ*G* = 0.0*k*_B_*T*; bottom: Δ*G* = 100*k*_B_*T*.

In Fig. 1 we report two simulation snapshots at different values of Δ*G*, showing the isodensity surface at half of the maximum density. Fixed ions on the channel surface and mobile counterions are depicted using blue and red spheres respectively. We apply the electric field along the *y* axis, parallel to the channel surface.

**Fig. 2:**
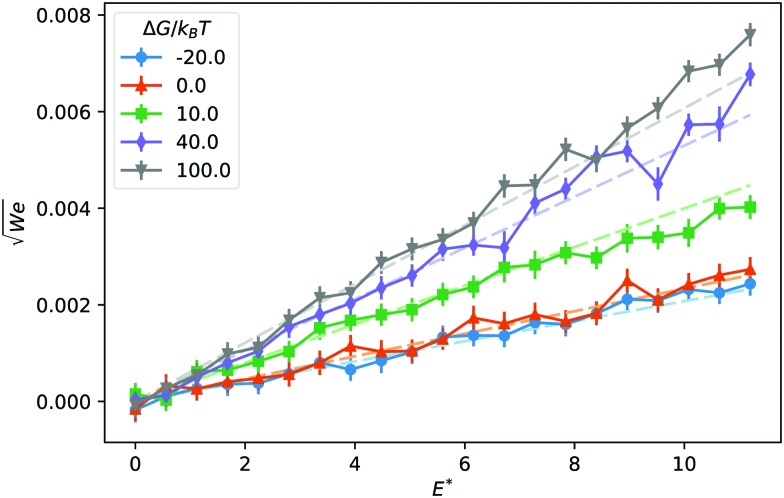
Reduced droplet terminal velocity as a function of the reduced electric field for different values of the solvation free energy Δ*G*. Dashed lines are the result of a fit to linear functions in the E* ∈ [0, 2.5] interval.

**Fig. 3:**
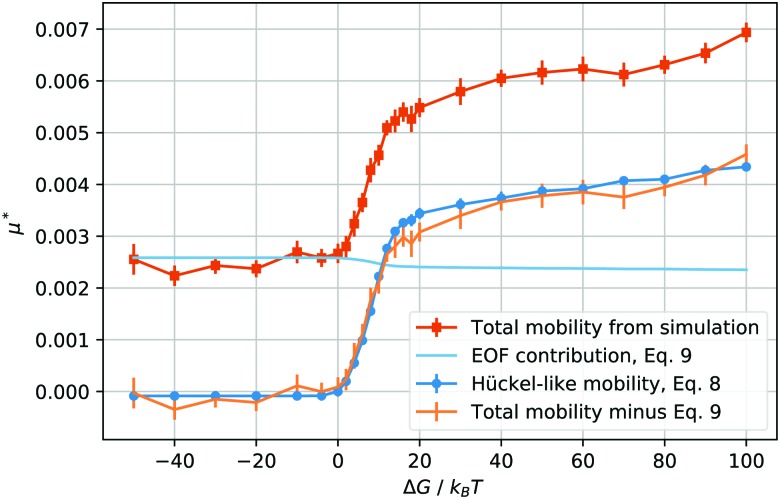
Reduced total droplet mobility μ* as a function of the solvation free energy Δ*G* of the counterions (orange squares); electroosmotic contribution, *μ*_eof_*, calculated using eqn (9) (light blue line); electrophoretic mobility, *μ*_ep_*, calculated from the droplet charge using eqn (8) (blue circles); electrophoretic mobility, *μ*_ep_*, calculated by subtracting *μ*_eof_* from the total mobility μ* (orange bars, no points).

The mobility *μ* of the droplet is defined by the terminal velocity *v*_t_ reached by the droplet in stationary conditions under the effect of an applied electric field *E*, as *μ* = *v*_t_/*E*. Since what we want to address is the problem of linear electrokinetic transport, we first checked, in which range of the reduced field E* the terminal velocity depends linearly on E* itself. In Fig. 2 we report the reduced terminal velocity *v*_t_*, which corresponds to the square root of the Weber number5
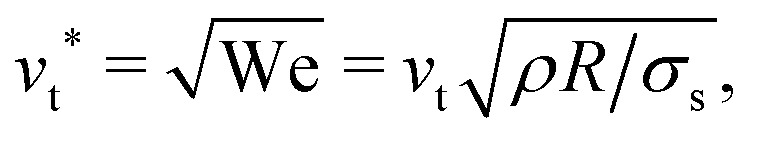
where *σ*_s_ is the surface tension (for the present choice of parameters, *σ*_s_ ≃ 0.41*k*_B_*T*/Δ*x*^2^, computed *via* the Laplace pressure jump), as a function of E* for different values of the solvation free energy Δ*G*. The terminal velocity is clearly linear at least up to values E* = 4. By using the result of the best fit to a linear function in the range E* = [0, 2.5], as shown in Fig. 2 using dashed lines, we report, in Fig. 3, the reduced mobility6
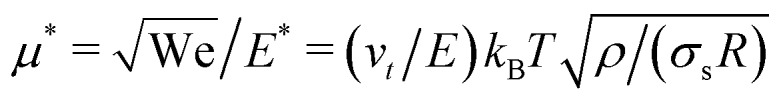
as orange squares.

When Δ*G* is negative, the droplet phase repels the ions and the mobility is slightly lower than in the neutral case, Δ*G* = 0. After we increase the solvation free energy to positive values, the mobility raises sharply, until the droplet transport enters into what seems to be a saturated regime. Intuitively, it seems straightforward to interpret the mobility values at Δ*G* ≤ 0 as mainly caused by the electroosmotic flow induced by the counterions. In fact, the droplet is in the middle of the channel, where the ion density is the lowest, and the electroosmotic flow is mainly generated in the high ion density regions next to the wall surface.

As soon as the droplet acquires some charge, when Δ*G* > 0, one could expect the onset of an electrophoretic behaviour. More precisely, for the droplet to move as a single charged object, the maximum solvation force needs to be larger than the electric force acting on each ion, or, Δ*G*/*k*_B_*T* > E*ξ/*R*, which, in our case, is valid for Δ*G*/*k*_B_*T* ≥ 1. In other words, given the parameters we have chosen, a free energy barrier large enough to prevent the thermal escape of the counterions will also prevent the electric field from stripping counterions from the droplet. Therefore, it makes sense to compute the charge of the droplet and to test whether its mobility at high solvation free energies is proportional to its charge *Q*, after removing the electroosmotic contribution.

If our droplet were a solid colloid, we would expect an electrophoretic behaviour of the Hückel type, since the ratio of the droplet radius to the screening length *κR* < 1. In this case, the mobility will be *μ* = *Q*/(6π*Rνρ*), if the slip surface is located at *R*. However, the droplet is not solid and it is therefore not correct to use the Stokes friction formula in the derivation of the mobility. In the limit of low droplet deformations,^60^ however, there is a simple solution for the friction coefficient *D*_visc_ = *F*/*v*_t_ of a droplet subject to a force *F*, as found by Hadamard and Rybczynski,^61^,^62^ and later rederived by Booth,^63^7
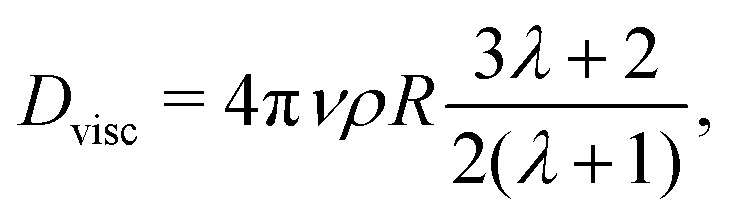
where *λ* is the viscosity ratio of the inner/outer fluids. In our case *λ* = 1, hence, *D*_visc_ = 5π*νρR*, where one readily recognises a Stokes-like expression with a coefficient of 5 instead of 6 for the solid sphere, which leads to a Hückel electrophoretic mobility

8 *μ*_ep_ = *Q*/(5π*ηR*)

for a charge distributed homogeneously in the droplet (we neglect conductivity contributions). However, not all charges in the droplet will contribute to the electrophoretic mobility. As a rough estimate, we consider the number of ions within the droplet radius *R* in the absence of solvation force to be always freely moving. Using this value of the charge to calculate the electrophoretic mobility, eqn (8), we report its rescaled value in Fig. 3 as blue circles.

The behaviour of the electrophoretic mobility contribution calculated this way resembles closely that of the total mobility, apart from a roughly constant offset. It is reasonable to believe that the origin of this offset is the electroosmotic flow, which drags the droplet along. From this perspective, the fact that the droplet mobility at values Δ*G* < 0 is lower than at Δ*G* = 0 can be interpreted as the droplet behaving as a charge hole in the local charge background, since the repelling solvation force is depleting the droplet of ions. Therefore, in order to interpret the observed mobility, we need not only the expression for the mobility, eqn (8), but also an estimate for the electroosmotic flow contribution. Here our Ansatz is that we can approximate the electroosmotic contribution as that in the middle of an identical channel in absence of the droplet. We take into account finite size effects by considering only the charges that are not trapped in the droplet as a source of the electroosmotic flow. Then an effective surface charge can be defined as *σ*_e_′ = *σ*_e_ – *Q*(Δ*G*)/*S* and used to compute the Gouy–Chapman length, and, in turn, the approximation for the electroosmotic contribution to the mobility^55^9
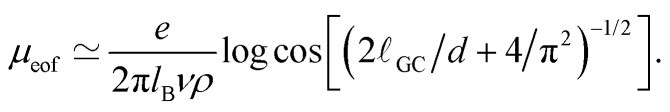



The electroosmotic contribution *μ*_eof_ is reported in reduced units as a continuous line in Fig. 3 and over the whole range of Δ*G* simulated, the contribution changed by 17% of its maximum value. With all contributions at hand, we are now ready to check that it is possible to express the droplet mobility as

10 *μ* ≃ *μ*_eof_ + *μ*_ep_

with the expressions for the electrophoretic and electroosmotic contributions given by eqn (8) and (9). In Fig. 3 we report the difference between the reduced mobility μ* and the reduced electrophoretic contribution *μ*_eof_*, which is found to be in good agreement with the reduced electrophoretic contribution *μ*_ep_*.

## 4 Conclusions

The problem of transport of droplets under microscopic confinement is relevant for a large number of microfluidic applications. The presence of counterions released in solution from the confining surfaces, by charging the fluids, opens the possibility of controlling droplets using electric fields. Even in the linear electrokinetic regime, where the potential drop across the droplet is smaller than the thermal energy, understanding the droplet transport is far from a trivial task, because of the superposition of the electroosmotic flow contribution and the electrophoretic mobility. The two contributions cannot be easily separated experimentally, a well-known issue in the context of colloidal electrophoresis, which has never been tackled in the case of fluid droplets. Computer simulations provide the possibility to disentangle these two phenomena by giving direct access to the droplet charge. Using a combination of on- and off-lattice simulation methods we modelled the transport of a droplet in a microfluidic channel as a function of the ions' solvation free energy difference between the dispersed phase and the medium. The solvation free energy of the ions is, in the present approach, an independent parameter. This allowed us to study the transition from the electroosmotic regime, where the counterions are dragging the medium, to the electrophoretic regime, in which the droplet moves as a charged object.

The present simulation does not take into account dielectric mismatch between the two fluids (nor with the confining medium). The electrophoretic mobility, however, as O'Brien and White pointed out,^64^ should not depend on the dielectric mismatch between solvent and, in their case, the colloid, but only on the zeta-potential. In other words, dielectric boundary forces should not influence the mobility in the linear regime. The same applies to droplets or bubbles.^9^ In our case, a change in dielectric mismatch would alter the Bjerrum and the Gouy–Chapman length. In practical terms, one would need to take care of using the correct values for the Bjerrum length (which depends on the dielectric permittivity) and of the surface charge (which should include polarisation charges).

In order to interpret the simulation results, we formulated a model for the total droplet mobility, which combines the electrophoretic contribution in the Hückel limit and an analytical expression for the electroosmotic flow. It is worth noticing that the well-known expression for the electrophoretic mobility of droplets by Baygent and Saville,^9^ based on the solution proposed by Booth^63^ reduces, in the zero salt concentration limit, to the Stokes case *Q*/(6π*ηR*). This expression, however, applies only to uncharged droplets,^63^ therefore, not to the present case, where the Hadamard–Rybczynski solution holds.

In summary, we have applied a mesoscopic simulation technique to the study of droplet transport in microfluidic channels. The simulation results support the interpretation of the total mobility as the superposition of an electroosmotic and an electrophoretic terms. The expression we proposed relates the total droplet mobility to its charge, as a function of known parameters such as fluid viscosity and channel surface charge density. This expression could be of practical relevance for the determination of individual droplet charge in microfluidic devices.

## Conflicts of interest

There are no conflicts to declare.
